# Transactions of the Medical Society of the County of Kings

**Published:** 1866-01

**Authors:** 


VOL. V. JANUARY, 1866. No. 6.

### ART. I—*Transactions of the Medical Society of the County of Kings. Specimens and Cases.*

### MONTHLY MEETING, JULY, 1865.

### *Tuberculous Disease of the Foot.*

Dr. Stiles presented a diseased *foot*, removed from a woman, aged twenty years, and an inmate of the Kings County Hospital for four months, having been for one year previously, under the care of a physician in Brooklyn. Shortly after admission an intense inflammation set in, in the affected part; this, however, gradually subsided, but not until *sinuses* had formed, which communicated with cavities in the foot. The subsequent treatment was directed principally to the general system, but the patient became weaker, and amputation was decided upon. On examining the amputated foot, found a *tuberculous* disease of the astragalus, a disorder that occurs but rarely in that bone. In the anterior portion of the astragalus and os calcis was a cavity lined by false membrane, and connecting with this were numerous sinuses all over the foot. In this case had a probe been introduced, no dead bone could have been felt, owing to the presence of this false membrane. There was no disease of the tibia. The suppuration was so profuse that the patient would have died had not amputation been resorted to. Syme's operation was talked of in this instance, but it was thought inapplicable.

Dr. Enos briefly referred to the case, and thought that Syme's operation might have been successful.

Dr. Wm. Otterson remarked, that in civil life Peregoff's operation, in these cases, might be resorted to with success; but among soldiers, whose constitutions were more or less feeble, and their vitality impaired, it rarely succeeded. In the present instance, the patient being so weak, and Syme's operation being so low down, he thought it would not be successful; the parts would not heal.

Dr. Minor thought that Syme's operation might have been first resorted to, and then if the tissues were found diseased, the operation might be performed higher up. If success attended the first operation, the patient would have a larger stump, and if the peroneal artery were left long, it would prevent a great deal of sloughing.

*Rupture of the Heart.*

Dr. Speir presented a specimen of rupture of the heart. On opening the pericardium a large clot was found enveloping the heart, and on removing this, a little blood spirted out of a small opening, which communicated with the left ventricle. The heart was hypertrophied and fatty, and at the point of rupture the wall was very thin, and exceedingly fatty. A few of the columnæ were ruptured, and there was calcification of the coronary artery. Atheromatous deposits were also found.

Dr. Enos stated that he had been in the habit, for some time back, of tapping this man for hydrocele, in his office. His attention had never been called to any trouble about the heart. About a week ago he was sent for in haste, but on arrival found him dead. On enquiry found that the man had come home that evening, complaining of feeling a little chilly, and soon afterwards fell down and expired. He had been lifting a little that day, and complained of feeling tired.

The question here recurs, did the *lifting* cause this small rupture? He thought it strange that this man should have had such an amount of disease of the heart, and still not complain of it.

Dr. Stiles referred to a similar case reported by Cruvellier, where there was a large mass of fat on the heart, giving it a yellowish appearance.

---

### MONTHLY MEETING, OCTOBER, 1865.

*Valvular Disease of the Heart.*

Dr. Stiles presented a specimen of endocardial trouble. The patient had been suffering for six months from disease of the heart, and when admitted into the Kings County Hospital, the dyspnœa was very distressing. There was slight swelling of the lower extremities. There was dulness on percussion for the space of three inches above the nipple, and auscultation revealed the double bellows murmur, but no rasping sound. The murmur was very slight with the first sound of the heart, but very distinct with the second. The man had not had any prior rheumatism. He died suddenly on the 14th instant. On post mortem found a perforation of one of the semilunar valves; to this cause can be attributed the bellows murmur. One of the semilunar valves was enlarged, the others small.

*Typhoid Fever.*

Dr. Stiles also related the following case: About three weeks ago a man was admitted into the Kings County Hospital with typhoid fever, and had so far recovered as to be able to go about the ward. In returning from the water closet last night he fell down, and soon afterwards expired. On post mortem found a profuse ulceration of the colon in the right hypochondrium, and a small perforation of the intestine. There was a large amount of yellow fæcal matter in the peritoneal cavity, and the abdomen was tympanitic. The ulceration in this case had not been confined to Peyer's plates. Between the ulcerations the membrane was greatly inflamed.

Dr. Hutchison presented a specimen of senile gangrene of the foot and leg, from calcification of the arteries. (Notes by Robert Newman, M. D.)

Joseph Stephenson, aged 64, a native of Ireland, for 24 years a resident of this country, was admitted to the Long Island College Hospital, Brooklyn, March 10, 1864, with dry gangrene of the left lower extremity. He stated that he had always been healthy, but for the past four years very intemperate. On the 20th of February ult., while returning home intoxicated, he fell several times, once hurting himself so severely that both he and those who came to his assistance supposed the leg to be broken. On the following day he noticed a slight contusion on the left leg an inch and a half above the external malleolus, and felt a numbness and coldness in that foot. When admitted to the hospital March 10th, the entire foot and a small portion of the leg were black, dry and shriveled, resembling closely the appearance presented by a dried mummy. Its condition at that time is well shown in the specimen. The gangrene extended somewhat higher upon the anterior than upon the posterior portion of the leg, and formed a distinct line of demarcation. The leg was enveloped in cotton batting, supported by a bandage. He was given beef-tea and the most nourishing food, ale and ℥ ij of whisky per diem with opiate.

On the 18th the line of demarcation being completely and distinctly defined, and the general health of the patient not being likely to improve, it was decided on consultation to amputate the leg. This was done by Dr. J. C. Hutchison, Professor of Surgery in the presence of the medical class. The leg was amputated at the point of election, four inches below the knee, by the flap operation. There was less than an ounce of blood lost, the only hæmorrhage being from the cutaneous vessels. The large arteries were in a state of calcification, and so completely blocked with lymph as not to bleed. This state of the arteries gives sufficient explanation as to the cause of the gangrene. Though scarcely necessary, a ligature was applied to the anterior tibial artery. The flaps were joined together by silk sutures and adhesive strips. The flaps and limb were cold at the close of the operation. Hot sand bags were placed about it until the limb regained its normal temperature. March 28, gangrene set in at two points on the anterior flap. Pulse 100 and feeble; patient taking food and stimulants freely. On the 30th the gangrenous portions of the flap began to slough, laying bare the tibia. The wound continued to slough for ten days following. The prostration gradually increasing until the afternoon of April 13, when he died, twenty days having elapsed since the operation.

From a post-mortem examination held the following morning, it appeared that the gangrenous slough had extended nearly to the knee. The femoral artery and sheath were surrounded by masses of fat intermingled with calcareous deposit. When first removed, it had the appearance of being badly injected with plaster of paris, and was, as is shown by the specimen, in a state of calcification.

In the specimen the arteries of the leg have been dissected from the point of amputation nearly to the gangrenous portion, and present the appearance of mere bony tubes or cords. The anterior tibial was completely calcified and occluded, and the posterior tibial nearly so. The peroneal was much enlarged, was also diseased, but the most perfect. The left ventricle of the heart was slightly hypertrophied, the aortic semilunar valves thickened, one segment gaping widely. Atherometous and calcaneous deposits were found in two valves; the aorta was normal. There was no lesion in the other organs.

It is evident that the gangrene was caused in this case by the obstructed condition of the trunks of the vessels, which existed for some time before the death of the part took place. The existing cause was probably the slight inflammation produced by the contusion before referred to, which disturbed the balance of the circulation in the already weakened part to so great an extent that gangrene ensued.

*Dysentery.*

Dr. Stiles presented three specimens, selected from fifteen cases of dysenteric affection. In general he found that the ulcerations had destroyed the mucous membrane down to the cellular tissue. Sometimes the membrane was perforated like a sieve; at other times it looked as if shot were scattered under the membrane. In one case there was no ulceration, but the folds of the membrane were sphacelated. When these folds slough, the case terminates fatally, and in his opinion a few hours is sufficient to convert a curable into an incurable case. The thickness is in the muscular coat, and in the cellular tissue, and not alone in the sub-mucous cellular tissue. There was no lack of secretion of bile, but the excretion of it was deficient.

Dr. Reese inquired how it was that death took place so suddenly from the perforation of the bowel in the case of typhoid fever?

Dr. Bell inquired why sphacelus of the folds of the mucous membrane should necessarily make a case incurable? He thought there might be very extensive ulceration of the mucous membrane, and possibly even of the muscular fibres of the intestine, and recovery still take place.

Dr. Enos agreed with Dr. Bell in regard to recoveries from extensive ulcerations of the intestines, and cited a case in which stricture of the rectum resulted from dysentery, yet the patient recovered. He looked upon the heart case as very interesting, from the fact of the semilunar valve being so large; and in the case of typhoid fever it seemed strange that the ulceration was so scattered, and not, as is usual, confined to Peyer's plates.

*Fibrous Tumor of the Uterus.*

Dr. Hutchison presented a fibrous tumor removed from the uterus. The lady was forty years old, had been married eight months, and was, until recently, in the enjoyment of fair health. About the first of May she was attacked with severe pain in the right side, and was seen soon afterwards by her family physician. In a week afterwards an abdominal tumor was first noticed. He saw her on the 20th of May, when the tumor was well marked, extending across the abdomen from the pubes to near the umbilicus. It was hard and somewhat movable, and could be traced down into the pelvis. A female catheter was introduced, but the handle had to be depressed, before the instrument would enter the bladder, indicating that the organ was spread out from the presence of this tumor. Hence the reason of the frequent micturation. In passing the finger into the vagina, the os was felt, but it was not definitely ascertained to be the os, until a sound was passed in about five inches. The tumor could also be felt in introducing the finger into the rectum. The lady had not menstruated for ten weeks, but there were no evidences of pregnancy. Was disposed to think, at the time of the examination, that it was a fibrous or ovarian tumor. He had been sent for to operate on the case, but thought it judicious not to interfere. She suffered considerably, and was only relieved by the administration of chloroform and subcutaneous injections of morphine. She died from nervous prostration.

On post mortem found the tumor to weigh 4½ pounds. It was examined under the microscope, and found to be fibrous in character. He did not think that the tumor was of such rapid growth as to attain its present size in three weeks; on the contrary, he imagined that it had been growing for some time before it was detected.

*Phosphatic Calculus.*

Dr. Hutchison also presented a phosphatic calculus removed from the bladder of a child, three years old, who had been suffering for one year previously, from the ordinary symptoms of this disease. The bilateral operation was performed; the child recovered. He also presented another calculus, removed from a child three years old. The child was suffering from tuberculous trouble in the system, and while under treatment the physician's attention was drawn to the urinary apparatus. He introduced a sound, and with considerable difficulty detected a stone. The patient was removed to the hospital, but died from the tuberculous trouble, and the stone and bladder were removed after death.

Dr. Stiles did not understand how the fibrous tumor of the uterus could have caused death in so short a time.

Dr. Hutchison thought it was owing to the complete nervous prostration, produced by intense pain and irritation.

Dr. Enos briefly referred to the case of stone, and remarked that the bilateral operation was the preferable mode of treatment.

*Blood Poison.*

Dr. Enos presented the left humerus of a boy, with the following history: He was in the enjoyment of fair health, and in the early part of the season was attacked with intermittent fever, from which he recovered. For the last two months though not robust, he enjoyed good health. On Wednesday, the 11th instant, he was seized with a chill, and complained of severe pain in the arm. He retained his consciousness, though suffering intensely from the local pain. Dr. Ingraham saw him on Thursday morning, and Dr. Enos had been sent for in consultation on the following Sunday. The boy had had febrile symptoms since Wednesday, and was a little obtuse in intellect—the arm was very much swollen and œdematous. On Sunday morning there was some evidence of vesication, and pitting was quite distinct on pressure. Leeches had been freely applied on Thursday, but with very little benefit. When he saw him he had a rapid pulse, the feet were inclined to be cool, the pupil was contracted, and the intellect rather obtuse, but still he could be roused. He seemed to be suffering a great deal from the pain in the arm, and the whole body seemed to be sore. The breathing was not particularly disturbed. A small incision was made through the affected part, from which serum escaped, and the pain seemed to abate in the arm. A warm poultice was then applied, and the tincture of iodine painted on the arm. He was ordered quinine, milk punch, beef tea, broth, etc. On Monday the symptoms were not more favorable; thought the pain had abated and the arm was less swollen, still the boy seemed no better, and appeared as if suffering from *blood poison.* Slight spasms supervened and became more frequent during the day. He died at four o'clock this morning.

On post mortem found ill-conditioned pus between the bone and periostium, and also in the tissues, but none was found in the shoulder or elbow-joint. The continuity of the periostium was gone. The left lung was normal, but small points of purulent deposit could be distinctly traced in its substance. The lower lobe of the right lung was engorged and adherent, and in its substance were small deposits of purulent matter. As we had come unprepared to make a post mortem, the brain could not be examined. In conclusion he would add that there was no jaundiced condition of the skin, and no marked *rigors* during the continuance of the trouble. He thought the affection in the arm was a local manifestation of the general trouble.

Dr. Stiles remarked that he had also seen a similar case with Dr. Ingraham, but in this instance there were *rigors*, and intense pain in the calf of the leg, and a jaundiced condition of the skin. The whole cellular tissue of the muscles of the leg was filled with ill-conditioned pus. On the front of the arm was an ecchymosed spot.

Dr. Bell presented a specimen of fatty degeneration of the liver. The patient was taken sick about eight months ago, in California, where he was treated for Bright's disease of the kidneys. He returned home about seven weeks ago, and was attended by Dr. Catlin, who could not say positively that he was suffering from that trouble. The man was tapped for ascites. On post mortem found the kidneys were undergoing fatty degeneration, but not to a fatal extent; the liver was fatty, and only weighed 1¼ pounds.

### MONTHLY MEETING, DECEMBER, 1865.

*Gun-shot wound of the Brain; the ball traversing both hemispheres; death six months later by Scarlatina; specimen presented by Dr. John C. Hutchison.*

Lydia Lista, a little girl aged seven years, walked to my office July 4, 1864, with her mother, who stated that her daughter had been injured by a buck-shot fired from a toy cannon by her brother while at play. She fell to the ground immediately on receipt of the injury, and vomited soon afterwards. I introduced a probe into the external wound, which was situated on a level with the top of the right ear and half an inch posterior to it, expecting from the appearance of the child that the shot had not punctured the skull. The probe, however, entered the brain substance and passed in about four inches. There was no opening on the opposite side of the skull. I expressed an unfavorable prognosis and sent the patient home, requesting the mother to call her family physician, Dr. Isaac H. Barber.

I did not see the child again, but Dr. B. has informed me that there were *no symptoms* of any description indicating injury of the brain except some slight vomiting, which continued for two or three days. No treatment was deemed necessary except rest, and she soon appeared as well as ever. She remained in good health, going to school and playing as other children until January, 1865, when she was attacked with scarlet fever and died of that disease on the 17th of that month. She had no symptoms indicative of disease of the brain during her last sickness.

On the day after her death a post mortem examination was made by my pupils, J. C. Goodridge, jr. and J. H. L. Elmendorf. Hearing of the death but a short time before the funeral, and the family positively refusing an examination, being in an adjoining room, made it necessary to conduct it with the utmost secresy and as expeditiously as possible. The brain being removed was brought to my office for examination. The specimen shows by four slightly depressed cicatrices that the ball entered the posterior lobe of the right hemisphere, near its juncture with the middle lobe, and emerging from this it crossed the longitudinal fissure, entered the left posterior lobe and made its exit from the brain upon the opposite side; then traversing the cerebrum from right to left in a direction backward and upward. The condition of the brain at the points of entrance and exit of the ball were normal. The membranes were healthy. Finding the ball had passed entirely through the brain substance had not fallen back into the original track, and could not be found by such incisions as would not injure the specimen, we assumed that it was imbedded in the skull near its point of exit from the brain, and that in the necessary haste of the examination it had been overlooked.

On examining the brain to-day, December 27, 1865, Mr. Goodridge detecting a point of unusual hardness and a corroded substance, found the ball imbedded in the substance of the brain near the surface an inch and a half in front and half an inch below its point of exit from the left hemisphere. I suppose that after traversing the cerebrum the ball struck the skull of the opposite side and rebounding lodged in the brain at or near where it was at last discovered. The specimen has been in a preparation of corrosive sublimate and alcohol for nearly a year, consequently the ball was much corroded. The portion remaining presents an irregular angular appearance, and is about half its original size.

*Recapitulation.*—We have then a girl seven years old injured by a *buck-shot penetrating the cranial cavity.* The child *walking* to the office and back home. A *probe passed* into the track of the ball *four inches. No brain symptoms appearing* except slight vomiting, which lasted but two or three days. *Entire recovery*, the child going to school and playing as other children. Subsequent death from another cause and a post-mortem examination revealing that there had *been no disease of the brain;* that the *ball* had *traversed* the posterior lobes of both hemispheres of the cerebrum, and rebounding had lodged in the brain substance, where it had remained with impunity, causing no inconvenience, and had become almost "a forgotten thing."

---

MORTALITY IN NEW YORK.—The whole number of deaths in the city of New York in 1864 was 25,645, an increase of 449 over the previous year, and of 4,401 over 1862. There were 1,425 deaths from typhus and typhoid fevers, and 394 from small pox and variola.

